# Making the Environment an Informative Place: A Conceptual Analysis of Epistemic Policies and Sensorimotor Coordination

**DOI:** 10.3390/e21040350

**Published:** 2019-03-30

**Authors:** Giovanni Pezzulo, Stefano Nolfi

**Affiliations:** Institute of Cognitive Sciences and Technologies, National Research Council, 00185 Rome, Italy

**Keywords:** epistemic action, sensorimotor coordination, cognitive enrichment, sensorimotor enrichment, aliasing

## Abstract

How do living organisms decide and act with limited and uncertain information? Here, we discuss two computational approaches to solving these challenging problems: a “cognitive” and a “sensorimotor” enrichment of stimuli, respectively. In both approaches, the key notion is that agents can strategically modulate their behavior in informative ways, e.g., to disambiguate amongst alternative hypotheses or to favor the perception of stimuli providing the information necessary to later act appropriately. We discuss how, despite their differences, both approaches appeal to the notion that actions must obey both epistemic (i.e., information-gathering or uncertainty-reducing) and pragmatic (i.e., goal- or reward-maximizing) imperatives and balance them. Our computationally-guided analysis reveals that epistemic behavior is fundamental to understanding several facets of cognitive processing, including perception, decision making, and social interaction.

## 1. Introduction

There have been rapid and impressive advancements in machine learning and artificial intelligence (AI), which have led to the solution of challenging games like chess or go [1]. Yet, most of these recent successes are due to machine learning methods that work particularly well in environments that are both deterministic (i.e., actions always have the same effect) and fully observable (i.e., current observations unequivocally inform an agent about its current state or situation). 

Determinism and full observability are met in games like chess or go but are rare in the real world. Living organisms evolved to make decisions and take action in the real world on the basis of significant uncertainty about their action outcomes and partial information about the environment in which they operate. Here, we focus in particular on the well-known “aliasing” problem, i.e., the fact that an agent can observe the same stimulus in different situations, and therefore the current observations do not unambiguously inform an agent about its current state or situation.

**Figure 1:**
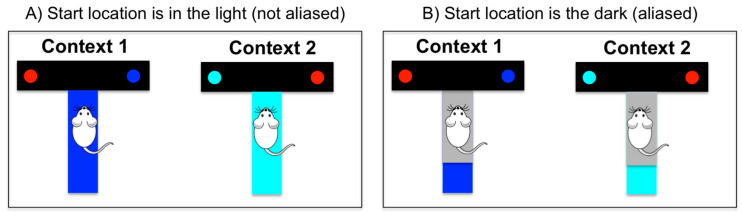
A simple T-maze decision problem. An artificial agent is located in a start position and can be in one of two contexts: a blue maze, in which reward (blue circle) is located at the right branch; or a cyan maze, in which reward (cyan circle) is located at the left branch. (**A**) When the start location is in the light, the agent can observe the color of the maze and determine in which context it is and its optimal policy (i.e., turn right in the blue maze and left in the cyan maze). (**B**) When the start location is in the dark, the agent observes the same ambiguous (grey) color. Based on this cue, it cannot determine in which maze it is or what its optimal policy is.

Imagine, for example, an agent is at the start position of a T-maze in which only one of the branches is baited with a reward; see Figure 1. The agent knows that it can be in one of two possible “contexts” (e.g., a blue maze or a cyan maze), and that reward locations are different for each context (e.g., reward is in the left branch in the blue maze and in the right branch in the cyan maze). If there is light at the start location, the agent can observe the color of the maze, and this cue tells it immediately in which context it is. In this condition, the agent can easily learn a behavioral policy that maximizes reward, i.e., a policy that turns right in the cyan maze and left in the blue maze. However, if there is no light at the starting location, there is a problem of aliasing, i.e., the agent receives the same observation (e.g., that it is dark) in both mazes. Thus, it cannot distinguish in which context (or maze) it is, nor can it determine an optimal policy.

How do living organisms address these problems? Here, we present a conceptual analysis of the importance of epistemic policies and sensorimotor coordination in conditions of limited and uncertain information. We use examples from previously published computational studies [2,3,4] and contextualize them within a general perspective on “how to make the environment and informative place” during adaptive behavior.

Our analysis distinguishes two families of computational solutions to the problem of aliasing, which appeal to notions of “cognitive enrichment” (i.e., making the agent’s internal representation richer and more informative about the current situation) and “sensorimotor enrichment” (i.e., making the next stimulus more informative about the current situation), respectively. We show that, despite their differences, these two methodologies are not mutually exclusive, and both appeal to a common notion of informative or epistemic actions (or action sequences, i.e., policies). Epistemic actions or policies have (explicit or implicit) epistemic goals, such as changing one’s knowledge state or stimulus for informative purposes. Thus, they differ from purely pragmatic policies, which aim at maximizing rewards without additional informative purposes (as we discuss below, the distinction between epistemic and pragmatic policies is related to the distinction between exploration and exploitation in AI).

In the next two sections, we exemplify two computational models that realize “cognitive enrichment” and “sensorimotor enrichment” of the stimulus, respectively, and discuss in which ways their actions have epistemic components. Next, we discuss the similarities and differences between these two approaches and the importance of epistemic action for adaptive behavior.

## 2. Cognitive Enrichment of the Stimulus

One widespread approach in control theory and reinforcement learning consists of casting decision making and action (or policy) selection problems, such as the T-maze problem of Figure 1, in terms of a Markov decision process (MDP) [5]. An MDP is a formalism widely used in artificial intelligence and reinforcement learning to describe decision-making and control processes in discrete time steps. At each time step, an agent is in a given state *s* (from a finite set of states *S*) and has to select an action *a* (from a finite set of actions *A*). After executing the action, two things happen. First, the agent moves to a state *s′*; the probability of doing a transition from *s* to *s′* after executing *a* depends on a so-called “transition function” (which may or may be not known to the agent). Second, the agent may receive a reward *r*; reward delivery for a transition from *s* to *s′* after executing *a* depends on a so-called “reward function” (which may or may be not know to the agent). The general goal of an agent (e.g., a reinforcement learning agent) who addresses MDP problems is to find a policy—i.e., a function that specifies which action to select in which state—typically to maximize cumulative reward. Various methods have been developed over the years to achieve such goals, some of which can scale up to highly complex problems [5,6,7,8,9,10].

A fundamental aspect of MDPs that renders inference tractable is the so-called Markov property, or the idea that an agent selects a policy (or state-action mapping) based only on its sensory measurements without considering the “history” of previous stimuli and actions (i.e., the agent is memory-less). The MDP scheme functions appropriately in situations analogous to Figure 1A, where it is assumed that the agent can simply extract its state *s* based on the sensory stimuli its receives. However, it would be often ineffective in situations analogous to Figure 1B, when stimuli are ambiguous or aliased and cannot be directly used by the agent to infer in which state it is. In the latter case, even if an agent knows the (optimal) policy to deal with a given situation, it cannot use it because its current sensory stimulus (e.g., the grey stimulus it observes in the start location) does not fully specify in which state it is. However, in the latter cases, it is possible to extend the basic architecture of an MDP agent to include more internal variables—a method that we call here a “cognitive enhancement”.

There are various ways to do a “cognitive enhancement”. One possibility consists of relaxing the Markov assumption and assuming that the agent maintains some short-term memory of its history, e.g., of few previous sensory stimuli. This may help alleviate the aliasing problem, at least in some situations. For example, one can imagine that the agent shown in Figure 1 experiences the same maze multiple times. Even if it starts a given trial (t) in the dark, it might remember that in previous trials (t − 1, t − 2, t − n), it was in a blue maze and select a policy that is appropriate for this context. More broadly, an agent can distinguish perceptually aliased situations (e.g., in which of two perceptually identical states it is in) based on memory (e.g., by remembering where it comes from); see [11] for an example connectionist system endowed with a short-term memory, which can address aliasing problems. The memory-based approach, however, requires solving a number of additional problems, such as deciding which stimuli to maintain in memory and when to update the memory [12]. More broadly, learning long-term dependencies (e.g., from a stimulus acquired at time t to an action to be selected at time t + n, with n being a big number) can be computationally challenging and require large amounts of data.

Another approach consists of considering formal extensions of the MDP scheme that explicitly deal with partial observability. The most widely used extension of the MDP scheme that explicitly considers non-observable processes or hidden states is called the partially observable Markov decision process (POMDP) [13]. It assumes that an agent cannot directly observe its state *s* (which is therefore called hidden or latent), but it can infer it based on the sensory observations *o* it receives (as well as its knowledge of its previous state and action). To continue our T-maze example, one can assume that the agent’s environment includes two hidden states or contexts (blue and cyan mazes). While the agent cannot directly observe its current state (i.e., in which maze it is), it can infer it based on the fact that each maze (blue or cyan) generates some specific sensory observations (blue or cyan stimuli, plus rewards) at some specific locations. The latent state inference may require that the agent maintain a belief (i.e., a probability distribution) about the hidden state *s* it may occupy and continuously updates it based on novel sensory observations *o*. 

A POMDP agent is “cognitively enriched” compared to an MDP agent, as it is endowed with (beliefs about) hidden states, which are more sophisticated than the state representation of an MDP agent (which maps directly to its sensory observation) or of sequences of stimuli in memory. Importantly, by knowing its hidden state or context, a POMDP agent can select an optimal policy (which would have been impossible just based on observation). For example, in our T-maze example, an agent that knows whether it is in the blue or the cyan maze can readily select an action that reaches the reward location. However, this requires a more complex decision process compared to an MDP agent. While a standard MDP agent would select a policy based on its current observation, a POMDP agent has to firstly infer in which hidden state or context it is (e.g., am I in the blue or the cyan maze?) and then select a policy based on the hidden state (i.e., since I inferred that I am in the blue maze, I go to the right). The resulting POMDP decision process is typically challenging or even intractable in real-world situations with a large state space, but several approximate solutions have been devised that render them more tractable [13].

More importantly, this hidden state inference lends itself quite naturally to forms of epistemic actions (or policies) that explicitly aim at collecting relevant information or reducing uncertainty before a choice. To understand why this is the case, we have to consider how hidden states or contexts are estimated in recent approaches to solving POMDPs, such as model-based reinforcement learning, planning-as-inference [14], KL control [15], and active inference [3,16]. These approaches use a model-based probabilistic scheme for representing and updating hidden states. The scheme is called model-based because it includes an internal (generative) model that describes the probabilistic transitions between hidden states conditioned on actions (e.g., if I am in the start location of the blue maze and I go right, I will be in the right branch of the blue maze) and the contingencies between hidden states and observable outcomes (e.g., if I am in the right branch of the blue maze, I expect to see a blue reward). In a probabilistic setting, hidden states are variables that are part of the agent’s internal model of the task. These are expressed as beliefs (technically, probability distributions) over variables of interest, such as the current location (being in the start location, in the left branch, etc.) and context (being in the blue or the cyan maze).

Beliefs about hidden state or context need to be continuously updated to be useful for decision-making. Importantly, despite the fact that hidden states cannot be directly observed, they can be inferred based on prior knowledge (which is part of the generative model) and observations using standard Bayesian inference. Typically, a belief is updated by integrating (in a Bayes-optimal way) the previous belief (at time t − 1) and sensory observations (at time t), such as in a Kalman filter [17]. Importantly, agents can also use more active strategies for perception and belief updating—they can select (epistemic) actions that are particularly informative and help disambiguating amongst alternative belief states [18]. A real-world example is coastal navigation, in which a person navigates towards a known landmark (e.g., the coast) to better self-localize, even if this is not the shortest path to the goal destination [19].

**Figure 2:**
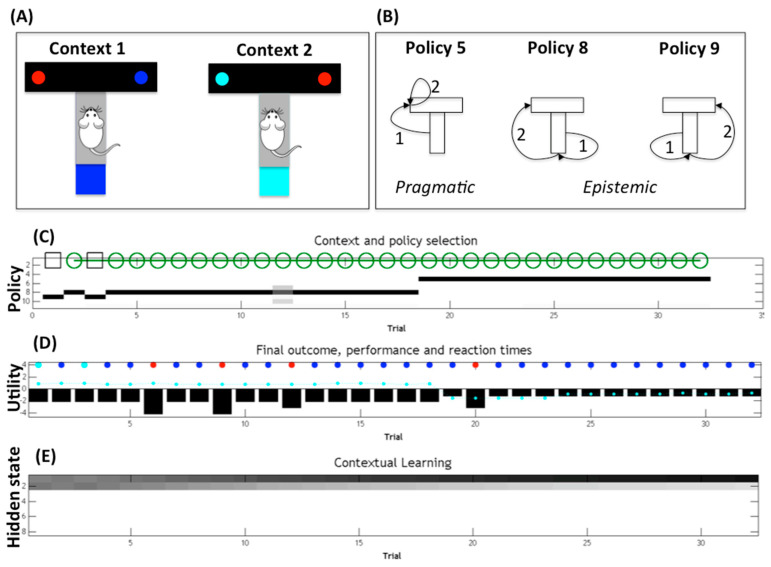
Epistemic versus pragmatic policies and belief-based versus stimulus-response (or belief-free) schemes. (**A**) A simple choice situation that includes four spatial locations (center, top-left, top-right, bottom) and two contexts (context 1 is blue maze and context 2 is cyan maze) for a total of eight (four by eight) hidden states. The start (center) location does not include any sensory cue that can disambiguate the current context. In context 1, the reward (blue circle) is located to the top-right, and a blue cue is located at the bottom. In context 2, the reward (cyan circle) is located to the top-left, and a cyan cue is located to the bottom. Red circles are not rewarding. (**B**) A simulated agent starts from the start location and has to select a sequence of two actions to reach the reward site, but it does not know the current context. Three example policies are shown: policy 5 (top-left, top-left) is a pragmatic policy that goes directly to the left location; policy 8 (bottom, top-left) and policy 9 (bottom, top-right) are epistemic policies that go to the informative (bottom) location before reaching one of the two reward locations. (**C**) Results of an example simulation using the belief-based scheme of active inference [2,20]. The panel shows the sequence of contexts (context 1 is represented by the green circle and context 2 by the black square) that the agent encounters for each trial and the policies it selects (horizontal lines); this specific sequence was selected beforehand for illustrative purposes. Grey scale colors indicate probability of selecting policies (black is high probability, grey is lower probability). There is a clear transition between epistemic policies 8 and 9 to the pragmatic policy 5 at trial 18, when the agent has resolved its uncertainty about its current context. (**D**) Outcomes (cyan and blue dots are rewards, red is no reward; note that outcomes are stochastic), performance (expected utility, with zero as maximum value), and reaction time for each trial. Reaction times represent the time to complete the choice and are based upon the processing time in the simulation [3]. (**E**) Agent’s belief (in probabilistic terms, i.e., probability distribution) about its starting state. The values 1 and 2 indicate the start location in context 1 and 2, respectively. Note that the agent increases its belief about being located in context 1 across multiple trials (as it believes that the context only changes with a low probability). For further details on the simulation, see [2,20].

This example illustrates the fact that model-based agents need to constantly balance the two imperatives of exploration (select epistemic actions to collect more information about hidden states) and exploitation (select optimal pragmatic actions to reach goal states). Figure 2 shows the behavior of a model-based (active inference [2,20,21,22]) agent that solves the T-maze problem “in the dark” illustrated in Figure 1B. The simulation shown in Figure 2 spans multiple trials in which the agent has to collect rewards in one of two possible branches (right for the blue maze and left for the cyan maze). The agent’s internal model includes the belief that it can be in one of eight possible states—one of four locations (start, top-left, top-right, bottom) within one of two contexts (context 1 is blue maze and context 2 is cyan maze). At the beginning of each trial, the agent knows its initial location (start) but not its current context (which is hidden). The agent also knows that the bottom location of the maze is “in the light” and has colored cues (blue or cyan), which would immediately disambiguate its current context. Finally, it knows that the top-left and top-right locations (which may or may not be baited with a reward) are “absorbing”, i.e., the agent cannot leave them until the next trial.

The agent therefore has the choice between two conflicting policies (Figure 2B). The former (pragmatic) policy would go directly to one of the two reward locations, whereas the latter (epistemic) policy would firstly go to the bottom location to collect a cue that changes the agent’s belief state (i.e., reduces uncertainty about the hidden context) and then to the (disambiguated) reward location. The latter is called an epistemic policy because the first action (going at the bottom location) aims at changing the agent’s belief state and not achieving an external goal. 

The results of a sample simulation (Figure 2C,D) illustrate that a model-based (or belief-based) agent that has a notion of its contextual uncertainty selects epistemic policies (numbered as 8 and 9 in Figure 2C) to firstly reduce its contextual uncertainty; then, after disambiguating its current context (which is context 1 in the simulation), it goes to the correct reward locations with high confidence, collecting the reward (cyan and blue cues in Figure 2D) most of the times. Note that standard belief-free agents that have no notion of hidden state, or even model-based agents that have no notion of their contextual uncertainty, would have no incentive to resolve the uncertainty. These agents would then try to reach the reward location without resolving the contextual uncertainty first. In this example, they would select a (pragmatic) policy to go directly to one of the two reward sites (top-left or top-right), thus failing to collect rewards half of the time (at least in the first trials, before they are allowed to learn by trial-and-error); see [2] for a discussion. 

Of course, epistemic policies are only required when there is some residual uncertainty about the current context. The simulation (Figure 2C) shows that if the agent repeats the T-maze choice across multiple trials, it stops selecting epistemic policies after a sufficient number of trials. The key thing to notice is that the agent’s uncertainty about its current context is maximal during the first trial but decreases across trials as the agent accumulates evidence that it is indeed in context 1 (the agent’s beliefs about context 1 or 2 are shown in Figure 2E; darker means higher probability). The agent can accumulate evidence for its current context across trials because it maintains a memory of the previous context it encountered (and assumes that the context changes with low probability). This implies that if, at any given trial, the agent gathers evidence for being in context 1 (e.g., because it sees a blue cue or collects a reward in the left branch), it will start the next trial with lower contextual uncertainty, and so on. In the simulation shown in Figure 2, 18 trials are sufficient to remove any residual uncertainty about the current context (Figure 2E). It is at this point that epistemic behavior becomes unnecessary, and the agent can change its behavior from exploratory (to collect cues about the context) to exploitative (to go directly to the reward location). This behavioral change can be appreciated by noticing that, in Figure 2C, there is a passage from the (epistemic) policy 8 to the (pragmatic) policy 5 at trial 18. 

More formally, the active inference agent exemplified in Figure 2 scores the quality of each of its candidate policies and then selects amongst them by considering the (negative of the) expected free energy of all the future outcomes that each policy will produce [2]; see [16] for the full mathematical details. In the context of this article, what is most important is that the (negative of the) expected free energy is calculated as the sum of two factors. The former factor is called extrinsic value, and it measures to what extent the outcomes (expected to be) generated by the policy resemble the agent’s preferred outcomes (or goals). In the T-maze example, this factor favors policies that (are expected to) lead to rewarding locations. The latter factor is called epistemic value (or information gain), and it measures to what extent the outcomes (expected to be) generated by the policy will reduce uncertainty about the agent’s hidden states (i.e., information gain). In the T-maze example, this factor favors policies that (are expected to) solicit informative cues by going to the bottom location. Scoring policies according to the (negative of the) expected free energy of their outcomes thus permits an agent to jointly consider extrinsic and epistemic value. The ways these two factors are balanced during action selection (i.e., exploration-exploitation) depends mainly on the agent’s belief state and environmental statistics. Generally speaking, it is only when there is no residual epistemic value or information gain about the current context (e.g., in which maze am I) that extrinsic reward can be pursued efficiently (i.e., the agent can select the correct reward yielding branch of the maze). This implies that epistemic value will dominate the inference until the agent is sufficiently certain about its context. Rather, when the agent has resolved its contextual uncertainty or the environment is not ambiguous, per se, extrinsic value will dominate the inference. Furthermore, an environment where rewards are widely available (e.g., both branches of the T-maze are baited with a reward) or particularly high will also favor extrinsic value; see [2,20] for specific examples of how exploration and exploitation are balanced in active inference.

## 3. Sensorimotor Enrichment of the Stimulus

A second family of solutions to the aliasing problem consists of “enriching” the stimulus itself by selecting appropriate behavioral strategies. The rationale of this idea is that an agent’s actions contribute to shape its next stimulus (as much as a stimulus contributes to the next action selection [23,24]). If an agent selects actions in ways that carefully shape or enrich the next stimuli, it will never encounter the aliasing problem [25,26].

In other words, an agent can inject new information in its sensory measurements by carefully selecting its actions. In turn, this sensorimotor enriching strategy permits circumventing sensory aliasing without using short-term memories, hidden variables, or internal models. It is worth noting that the notion of “enrichment” cannot be taken literally; for example, an agent’s actions can also filter (or remove information from) stimuli. What is critical is that the actions alter the environment or the agent/environment relation in a way that permits the agent to later experience sensory states that include the information necessary to take the appropriate actions by reducing and eventually eliminating the need to rely on hidden states. 

Solutions of this type are consistently observed in experiments in which robots acquire their behavioral ability through a training process driven by an implicit performance measure [25,26]. The term implicit refers to the fact that the performance measure evaluates the extent to which the robot is capable of solving its task without specifying the characteristics of the behavior that should be produced by the robot. In a nutshell, the training process operates by introducing variations in the characteristics of the robot and by retaining or discarding the variations depending on whether they produce an increase or a decrease of the robot’s overall performance, respectively. Overall, this implies that the training process selects the simpler and most effective solutions that can be generated through random variations independently from whether such solutions rely on cognitive or sensory-motor enrichment of stimuli.

**Figure 3:**
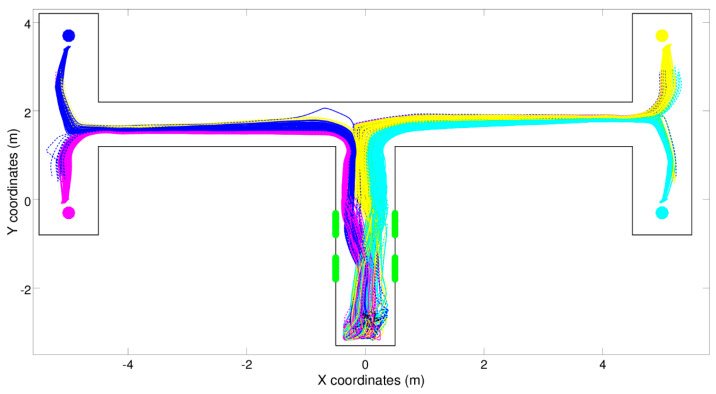
Trajectories produced by an evolved reactive robot during 300 navigation episodes. Full and dashed lines indicate successful and unsuccessful navigation episodes, respectively. The color indicates the corresponding context and target destination (magenta: left-bottom, blue: left-top, cyan: right-bottom, and yellow: right-top). The green bars indicate the location of beacons (only two beacons are present in each navigation episode).

Sensorimotor enrichment solutions can be exemplified by the experiments reported in [4], in which a simulated robot is situated in one of four types of double T-maze environments that differ in the position in which the food is located with respect to the central corridor (left-left, left-right, right-left, and right-right, see Figure 3). The type of the environment is indicated by the position of two green beacons located in the central portion of the central corridor, which can be perceived by the agent only in this part of the environment.

The problem admits a cognitive enrichment solution that relies on internal states (e.g., a first internal state that encodes the state of the beacons, which can be updated by the robot when it is located in the central portion of the central corridor, and a second internal state that encodes whether or not the robot passed the first junction that can be updated when the robot turns left or right at a junction) and a control policy that drive the robot forward in corridors and make the robot turn left or right at junctions depending on the value of the internal states. 

The problem, however, also admits a solution that relies on sensorimotor coordination and does not require internal states. Such a solution consists of reacting to the perception of the beacons in the central corridor and to the perception of the following stimuli in a way that ensures that the robots keep experiencing four different types of stimuli in the four corresponding types of the environment. This in turn enables the robot to turn in the appropriate direction at the first and the second junction on the basis of the current perceptual states only.

The latter solution is one consistently discovered by robots that are evolved for the ability to forage in a double T-Maze environment [4]. The robots are provided with a feed-forward neural network controller that includes: (i) infrared and visual sensors that enable them to perceive nearby obstacles and the beacons when the beacons are not visually occluded by walls, (ii) internal neurons that transform the current input pattern into an internal pattern, and (iii) motor neurons that determine the speed of the two robot’s wheels. The robots are initially placed in the bottom part of the central corridor. The initial position and orientation of the robots and the length of the corridors forming the T-Maze are varied randomly within limits at the beginning of every trial. The evolving robots are evaluated for multiple trials. 

The connection weights of the robots’ neural network are evolved [27]. This is realized by creating an initial population of robots with different randomly assigned connection weights and by generating new generations of better and better robots. New generations consist of mutated copies of the fittest robots. Mutations are realized by randomly perturbing a few randomly selected weights. Robots are evaluated for multiple trials, and the fitness of a robot is increased by one point for every trial in which the robot navigates directly to the food location. The position of the food and the corresponding position of the beacons are chosen randomly with a uniform distribution at the beginning of each trial during the evolutionary process and during post-evaluation. We used this approach since it imposes minimal constraints on the type of solutions that can be discovered by the robots and it can operate on the basis of a compact feedback. Full details are provided in [27].

Figure 3 shows the trajectories displayed by a typical reactive robot, i.e., a robot provided with a neural controller that does not have recurrent connections (i.e., does not have any memory) and consequently determines its motor reaction on the basis of the current state of the sensors only. As can be seen, the robot manages to navigate to the right destinations most of the time by turning correctly left or right at the first and the second junctions. The fact that the robot cannot perceive the beacons when it has to turn left or right at the junctions is solved through sensorimotor coordination. Indeed, evolved robots: (i) react to the beacons perceived while they travel in the central corridor by moving toward the left or the right side of the corridor depending on the state of the beacons, (ii) maintain those relative positions and orientations with respect to the corridor later on, and (iii) turn left or right at the first and the second junctions depending on their relative position and orientation with respect to the corridor. Rather than storing the state of the beacons in internal states for later use, the robots offload [28,29] the state of the beacons in the environment—more precisely, in the robot/environmental relation. The state of the beacons can then be inferred from the relative position of the robot in the maze, and such information remains available during the entire navigation phase. The robots provided with recurrent connections displayed qualitatively similar behavior; see [27] for details.

The strategy displayed by evolved robots can be described as a form of cognitive offloading in which agents “offload” their future intentions into the external environment, at least in part, rather than relying on internal states [30,31]. Strategies of this type are commonly observed in humans who use diaries to help themselves remember intended behaviors, place reminders in noticeable places, and interact with the world to reduce the computational load of subsequent information processing [30,31]. An example of the last categories include the strategy of catching a flying ball by adjusting the running speed so as to constantly maintain the relative angle between the agents and the ball [32]. More specifically, with respect to navigation decision at intersection points, the experimental results reported in [33] indicate that humans strive to make decisions long before overt responses are required.

**Figure 4:**
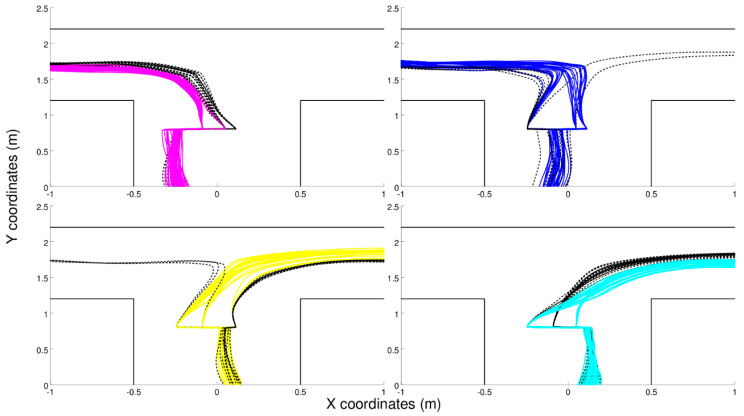
Trajectories displayed by an evolved robot with recurrent connections at the first T-junction during a post-evaluation test in which the robot is displaced over the left or the right of the corridor 40 cm before the junction. As with reactive robots, these robots arrive at the junctions by assuming different relative positions within the corridor in different contexts. The internal neurons enable the robot to re-enter in the trajectory they would have displayed if they were not artificially moved over to the left or the right.

Robots provided with recurrent connections display an ability to navigate to the current destinations also after being subjected to position-perturbations, i.e., after being moved on the left or on the right before junctions (Figure 4). Interestingly, these robots rely on a mixed solution based on the same type of sensorimotor strategy illustrated above combined with the extraction and use of internal states that enable them to reduce navigation errors and to recover from position-perturbations (to re-assume the appropriate position and orientation most of the time after a position perturbation).

**Figure 5:**
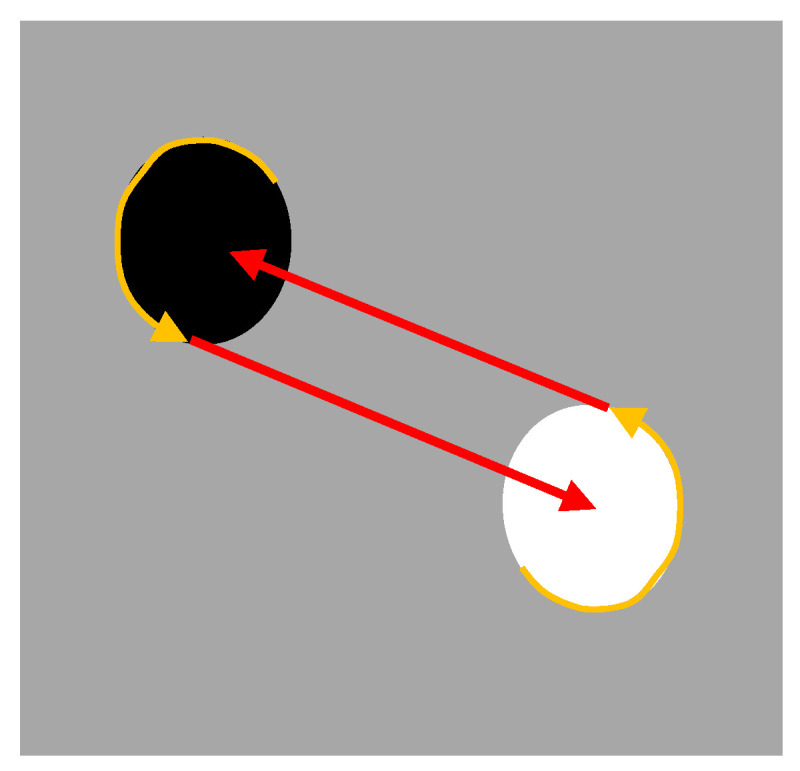
Illustration of the trajectories displayed by the evolved robots while they navigate from the nest to the foraging area and vice versa. During the phase shown in yellow, the robots move anticlockwise along the border of the area in which they are located until they visually perceive the other robot on the left side of their visual field. Then, during the second phase shown in red, the robots exit from their area and navigate toward the center of the destination area simply by continuing to move straight. The coordinated follow-border behavior thus enables the robot to infer and implicitly communicate to their pair robot the location of the center of their area.

Sensorimotor coordination also permits one to generate information that is not directly available for perception. A classic example of generation of information through action is constituted by the optic flow, i.e., the distribution of apparent velocities of movement of a brightness pattern in the image perceived by a moving agent. Optic flow can be used to generate distance information through the execution of a simple behavior that consists of, for example, straight movement. In other cases, the generation of the required information can require the execution of an articulated behavior. This can be illustrated by a second experiment [34] in which teams composed of two robots are evolved for collecting food in a foraging area and bringing the food to a nest area (the white and black circular areas shown in Figure 5). The relative position of the paired robot can be visually detected by distance, provided that it is located in the view range of the perceiving robot. The nest and the food areas, on the other hand, cannot be visually located. Robots only detect the color of the ground below them, i.e., only detect whether they are located over a food area or over a nest area.

The analysis of the robots evolved in these experiments shows how they exploit sensorimotor coordination to infer the position of the barycenter of each area, communicate this information to the pair robot, and use the communicated information to travel toward the barycenter of the other area, despite the fact that the center of an area is perceptually indistinguishable from nearby positions.

This is realized through the execution of a motor behavior executed by both robots, which consists of moving along the border of the area in which they are located anticlockwise until the pair robot enters in the right side of the perceiving robot visual field (Figure 5). This enables the two robots to exploit the geometrical characteristics of the environment in order to assume a precise position in their respective areas, i.e., to move along the border until they are situated on the right side of the center of their area with respect to the position of the perceiving robot. This in turn enables the two robots to infer that the barycenter of the destination area is located on the left of the companion robot, a destination that can be reached simply by moving straight from their current position and orientation as soon as the second robot appears in the right side of their visual field (Figure 5).

## 4. Discussion

In this paper, we exemplified two approaches—cognitive enhancement and sensorimotor enrichment—to solve sensory aliasing problems. The two approaches are fundamentally different, as the former renders an internal representation of the problem richer, and the second renders stimuli richer. However, in both approaches, agents realize the (cognitive or sensorimotor) enhancement by acting in informative ways. This implies that in both approaches, action selection does not only fulfill pragmatic imperatives (i.e., achieving goals) but also epistemic imperatives—the necessity to reduce uncertainty about a hidden state and/or render future stimuli more informative. This is an important point, as it reveals a deep similarity between two approaches that are often considered antithetic. 

The experiment with model-based agents (Figure 2) shows that cognitive agents constantly need to balance exploration and exploitation (or epistemic and pragmatic imperatives) during action selection. In other words, for a cognitive agent, a detailed internal model may not be sufficient to solve a task unless the agent also adopts active (e.g., hypothesis testing) strategies that render the models effective for action (or policy) selection [35,36]. This renders cognitive agents active entities that constantly seek evidence for their plans and their goals, rather than passive entities that try to mirror the external environment via their internal models [37,38,39,40,41]. Note that the model-based agent exemplifies the usage of an “explicit” model—it has an explicit, content-involving representation of its task, organized as an internal model (e.g., a map of spatial locations, reminiscent of hippocampal spatial codes [42,43,44,45]). Furthermore, it uses the model for explicit predictive inference (i.e., to predict future locations and associated rewards) that permits “scoring” the quality of candidate policies and ultimately selecting amongst them. While this agent architecture is compatible with current neurophysiological theories about map-based spatial navigation, the extent to which living organisms use explicit representations and inferential processes to support navigational (or other) choices is currently debated. In principle, similar results can be obtained using a more “implicit” model (e.g., a model that learns the contextually-appropriate policies by trial and error) that dispenses from explicit predictive processes; see [46] for a discussion of “explicit” versus “implicit” models. The empirical validity of architectures that use explicit or implicit models (or combine them) [47] has to be established case-by-case.

The experiments with evolved robots (Figure 3, Figure 4 and Figure 5) show that mechanisms like sensorimotor coordination, which are often disregarded in the study of cognitive processing, have to be considered part and parcel of it rather than just peripheral processes with little importance in regard to understanding how we perceive, think, or decide. Indeed, sensorimotor enrichment is consistently exploited in all experiments involving agents that are evolved or trained for the ability to solve a problem and are left free to determine the way in which the problem is solved independently from the nature of the problem; see, for example, [24]. In other words, sensorimotor enrichment constitutes a general strategy that can be applied to a vast range of problem domains. Focusing on sensorimotor coordination may help re-contextualizing classical views in cognitive science, such as the idea that perception, decision-making, and action can be described as separate processes that operate sequentially [48]. The experiments with evolved robots illustrate that such sequential structure is not (always) necessary, and the three above processes coexist throughout the duration of the task; see also [49,50]. Furthermore, the double T-maze simulation illustrates that tasks apparently requiring an explicit model or working memory can be solved efficiently using implicit models or reactive mechanisms [46], and that these mechanisms can act in concert with more cognitively sophisticated ones to steer adaptive behavior.

Cognitive and sensorimotor enrichment of stimuli may be considered alternative yet potentially complementary strategies for solving challenging problems where stimuli are limited or uncertain. The idea that living organisms maintain a detailed internal representation (or cognitive map) of the current situation to support adaptive choice over and above stimulus-response mechanisms can be considered foundational in the history of cognitive science, as exemplified by Tolman’s view about cognitive maps [51]. Importantly, living organisms may systematically adopt epistemic policies or information-gathering strategies to keep these maps (or much simpler implicit models, as explained above) “in register” and disambiguate amongst competing hypotheses [35,36], even if this comes at the expense of apparently suboptimal behavior, as in the case of navigation towards known landmarks in coastal navigation. Along similar lines, humans’ apparently suboptimal behavior during decision-making may result from the fact that they are simultaneously attempting to both optimize economic reward and to learn the causal structure of the task [52], the latter being an example of an epistemic action supporting model learning; see also [21,53]. Although the scope of these or other cognitive enrichment strategies is unclear, it is plausible that the presence of strong and easy-to-learn causal structure in the task at hand would favor them. 

Furthermore, there is compelling evidence that humans can productively “offload” some of the burden of cognitive processing and decision-making to the external environment. Experimental data collected by Gilbert [30,31] indicate that human subjects rely and benefit from sensorimotor enrichment, especially when they have a greater memory load, should perform other potentially distracting activities, or are older. Other potential advantages of sensorimotor enrichment include the possibility to extract relevant information over time to better filter out the effect of perceptual noise [54,55] and the tendency to trigger the execution of preparatory actions and favor the production of smoother behaviors [21].

Still, in other cases—probably the majority of cases—the best results can be obtained by combining cognitive and sensorimotor enrichment. An example is the experiment reported in Figure 4, in which sensorimotor and memory-based mechanisms operate synergistically. The combination of the two methods can produce better results than the usage of the two methods in isolation (for more details, see [4]). Another possible way to integrate cognitive and sensorimotor enrichments consists of starting from a model-based agent and augmenting it with the possibility to use sensorimotor strategies of the kind illustrated in Figure 3 to render their behavior more informative. A previous simulation showed that an active inference agent can learn epistemic policies (i.e., policies such as 8 and 9 in Figure 2) over time that are directly activated by stimuli rather than the usual predictive inference [3]. Similarly, the agent may acquire policies that solicit the most informative sensory cues for a given (later) decision, akin to the sensorimotor coordination strategy exemplified in Figure 3 and Figure 4, and use these cues (rather than predictive processing) for decision-making. From a formal perspective, endowing model-based agents with sensorimotor coordination mechanisms may allow transforming (some aspects of) a POMDP problem into a simpler MDP problem, which requires no internal state and would help alleviate the huge computational demands of POMDP schemes. These are, however, just examples of integrating cognitive and sensorimotor enrichment. In problems more complex than those reported above, the relationship between cognitive and sensorimotor enrichment might be much more profound and intimate, and it certainly deserves future investigations. 

## 5. Conclusions

We began with the question of how living organisms make decisions and select actions in conditions of limited and uncertain information and discussed the importance of selecting epistemic policies that disambiguate amongst alternative hypotheses or favor the perception of stimuli providing the information necessary to later act appropriately—hence favoring the “cognitive” and/or the “sensorimotor” enrichment of stimuli. Here, we offered two specific examples of epistemic actions or policies, but living organisms use many more strategies to modulate their behavior in informative ways. Some widespread examples are attention and active sensing dynamics, which permit an agent to sample its environment in ways that are especially appropriate for selecting actions or disambiguating hypotheses [35,36,56]. During problem solving, one can move objects around (e.g., rotate Tetris pieces before placing them) in order to unveil possible solutions that would be more difficult to identify by reasoning alone, thus rendering cognition more situated [57]. Another example is morphological computation, in which dynamical bodily properties can alleviate or even replace “central” (i.e., brain) computations [58]. Finally, as also illustrated in Figure 5, the active modulation of one’s own behavior can be informative not just for oneself, but also for others. Recent evidence indicates that actors engaged in a joint action strategically modulate their behavior to help their co-actors predicting or coordinating with them, thus engaging in a form of sensorimotor (non-verbal) communication that is guided by epistemic imperatives [59,60,61,62,63,64,65].

All these examples illustrate that epistemic strategies (sensorimotor and cognitive) are key in many domains of cognition (e.g., perception, decision-making), including social cognition (e.g., sensorimotor communication). It is thus important to fully recognize that oftentimes actions have both epistemic and pragmatic functions. The majority of the actions alter the relative position of the agents in the environment and consequently the stimuli that the agents will experience next. Therefore, actions playing a purely pragmatic role may better represent the exception than the rule.
